# An Emergency Medicine Virtual Clerkship: Made for COVID, Here to Stay

**DOI:** 10.5811/westjem.2021.11.54118

**Published:** 2021-12-17

**Authors:** Stephen Villa, Hannah Janeway, Kian Preston-Suni, Ashley Vuong, Ignacio Calles, James Murphy, Taylor James, Jaime Jordan, Andrew Grock, Natasha Wheaton

**Affiliations:** * University of California – Los Angeles, Department of Emergency Medicine, Los Angeles, California; † David Geffen School of Medicine at UCLA, Department of Emergency Medicine, Los Angeles, California; ‡ Greater Los Angeles VA Healthcare System, Department of Emergency Medicine, Los Angeles, California

## Abstract

**Introduction:**

Safety concerns surrounding the coronavirus 2019 pandemic led to the prohibition of student rotations outside their home institutions. This resulted in emergency medicine (EM)-bound students having less specialty experience and exposure to outside programs and practice environments, and fewer opportunities to gain additional Standardized Letters of Evaluation, a cornerstone of the EM residency application. We filled this void by implementing a virtual clerkship.

**Methods:**

We created a two-week virtual, fourth-year visiting clerkship focused on advanced medical knowledge topics, social determinants of health, professional development, and professional identity formation. Students completed asynchronous assignments and participated in small group-facilitated didactic sessions. We evaluated the virtual clerkship with pre- and post-medical knowledge tests and evaluative surveys.

**Results:**

We hosted 26 senior medical students over two administrations of the same two-week virtual clerkship. Students had a statistically significant improvement on the medical knowledge post-tests compared to pre-tests (71.7% [21.5/30] to 76.3% [22.9/30]). Students reported being exposed to social determinants of health concepts they had not previously been exposed to. Students appreciated the interactive nature of the sessions; networking with other students, residents, and faculty; introduction to novel content regarding social determinants of health; and exposure to future career opportunities. Screen time, technological issues, and mismatch between volume of content and time allotted were identified as potential challenges and areas for improvement.

**Conclusion:**

We demonstrate that a virtual EM visiting clerkship is feasible to implement, supports knowledge acquisition, and is perceived as valuable by participants. The benefits seen and challenges faced in the development and implementation of our clerkship can serve to inform future virtual clerkships, which we feel is a complement to traditional visiting clerkships even though in-person clerkships have been re-established.

## BACKGROUND

The coronavirus 2019 (COVID-19) pandemic drastically altered educational and residency application landscapes for emergency medicine (EM)-bound medical students by restricting in-person visiting clerkships.^1^–^5^ Visiting clerkships traditionally have been critical for EM-bound students.^6^ Medical students who are EM bound typically complete at least two EM clerkships to gain additional experience and explore varied training environments. Visiting clerkships also provide Standardized Letters of Evaluation (SLOE) – crucial to the residency application – and allow for reciprocal exposure between the student and program.^6^–^8^ In response to COVID-19 restrictions, many medical schools and residencies pivoted to online education and cancelled visiting rotations.^9^,^10^ With the loss of in-person visiting clerkships, a novel virtual curriculum was urgently needed to fill this void.

As an alternative to traditional in-person visiting clerkships, we created, implemented, and evaluated a virtual “visiting” clerkship with a focus on advanced and less commonly taught topics (ie, social EM and professional development). Based on the most recent recommendations, many institutions have removed restrictions on in-person rotations but continue to limit visiting rotations to one per student.^11^ Looking forward, the virtual environment creates a unique opportunity for programs to continue to meet their applicants more in depth, in addition to circumventing geographic and socioeconomic barriers often faced by students participating in traditional visiting rotations.

## CURRICULUM DESIGN

Rather than replicating a traditional clerkship virtually, we designed our curriculum to focus on advanced medical topics: social determinants of health; structural competency; and professional identity formation^12^, ^13^ by employing Kern’s method for curriculum development.^14^ We identified educational needs as institutional COVID-19 restrictions were released. We performed a needs assessment including data from our postgraduate year one class as near peers. Our topic list was further refined by consensus among the author group, which included a clerkship director, associate program directors, medical education fellow, and senior EM residents. We developed goals and objectives informed by the topic list and the additional goals of exposing students to our residency program and social EM, as well as advancing professional identity formation. Our traditional in-person sub-internship experience typically covers medical knowledge topics commonly seen in the emergency department as well as skills to help learners thrive while rotating in person. In addition to being vastly different from a traditional experience given that it would be delivered virtually, we felt that this rotation could possibly serve as an ideal environment to cover social EM and professional identity formation, topics that would benefit from minimal interruptions or competing pressures.

## OBJECTIVES

Curriculum goals included teaching advanced EM clinical knowledge, introducing social EM and professional identity formation, and providing exposure to our residency program. See Table 1 for course goals and objectives.

When choosing educational methods, we used the conceptual framework developed by Brown et al to maximize online learning and engagement. This framework encourages expectation management, learner engagement, and “nudging.”^15^ Our orientation outlined expectations, including asynchronous assignments and recommended norms for small group. We prioritized interactive teaching modalities and active learning to maximize engagement such as small-group learning among as well as our “Virtual Escape Room” (Appendix A) and simulation. Our small group, case-based discussions used a flipped classroom model, an effective and recommended modality for virtual instruction.^16^–^22^ All small-group facilitators were reminded of the best practices for online, small group teaching,^23^ which included use of introductions, learner-directed questioning to encourage equal participation, and “nudging” – reminders for learners to actively participate (See Appendix B).

Each virtual clerkship session was held on weekdays over two weeks for a total of 10 instruction days. The students were expected to complete various asynchronous learning assignments (estimated two hours daily) and attend four hours of Zoom (Zoom Video Communications, Inc, San Jose, CA) sessions daily. (See Appendix C for example schedule and specific content.) Cases from “Foundations of EM,” a national free, open-access online resource, were used to teach medical knowledge.^24^ Social medicine instruction was done using modules from the International and Domestic Health Equity and Leadership (IDHEAL) Section from the University of California, Los Angeles.^25^ Chosen modules included Language, Incarceration, Gender Identity, Race, and Homelessness, and assigned readings from those modules were delivered to learners via email. The Virtual Escape Room consisted of a tricyclic antidepressant overdose case, created by authors AV and TJ (Appendix A) with inspiration from another published escape room.^26^ The virtual simulation was carried out over Zoom, and cases from our traditional clerkship were used, which cover pediatric anaphylaxis, motorcycle trauma, hypothermia, and abdominal aortic aneurysm. The “Communities of Practice Panel” consisted of a panel of faculty/attendings who practice in different EM environments (ie, tertiary referral center, county, community, Veterans Administration, and critical access community). Many of these modalities have previously been highlighted as effective teaching modalities.^27^,^28^ Within the professional identity formation theme, learners read Carol Dweck’s “Mindset” to prepare for a book club-type discussion – a modality previously well-received by other learners.^29^ Finally, students were introduced to basic pedagogical techniques and practiced non-medical teaching sessions for their peers and faculty; feedback was provided.

Asynchronous assignments consisted of *Emergency Medicine Reviews and Perspective* C3 podcasts, free open-access medicine (FOAM) curated by the Academic Life in EM (ALiEM) AIR series, Foundations of EM “Frameworks,” and articles introducing topics of social EM.^24^,^25^,^30^,^31^ Asynchronous content was designed to correspond to daily synchronous content; specifics can be found on Appendix C. We made our virtual clerkship available to all fourth-year medical students applying into EM via the Visiting Student Application Service (VSAS) website and offered it twice during the 2020–2021 academic year.^33^ Given our predicted teaching resources, we estimated an ideal class size of less than 25 students per session. Ultimately, 26 students enrolled (nine in the first session, 17 in the second session). Attendance at all sessions was mandatory.

We recruited a group of residents and faculty to teach for a total of 24 lecturers and 26 small-group facilitators across both sessions. Facilitating the clerkship during the two-week session required one of three clerkship directors to be present on Zoom four hours per day, in addition to administrative tasks related to that day’s activities.

We assessed medical knowledge with a 30-item, peer-reviewed, multiple-choice test consisting of questions donated by RoshReview (Rosh Review LLC, Huntington Woods, MI), a commercial question bank company. RoshReview validates questions against real-world exam performance such as the in-training exam for EM residents.^32^ Questions were chosen by the course directors by a systems-based approach (ie, neurology, cardiovascular, etc) with the goal of choosing questions that were reflective of clerkship’s curriculum. Students did not have the ability to see the answers to the questions after taking the pre-test. Because the test was conducted at home, students theoretically could access open-access content in real time. Students completed the same medical knowledge test on the first and last days of the clerkship.^34^ We calculated mean scores and compared pre-and post-tests using a paired t-test, analyzed with the software statistical package Stata 16.1 (StataCorp, College Station, TX). Test scores had no bearing on final grades.

Each two-week clerkship contained five social EM sessions, which were assessed by an anonymous survey exploring previous experience with the topic and comfort with applying content learned to the clinical environment (Appendix D). This tool had been previously used in residency education by the IDHEAL group. It was developed by content experts after literature review to maximize content validity. We analyzed questions relevant to this clerkship. Participants were sent an online anonymous survey after each of the five sessions. In total, we sent a total of 130 surveys (five surveys per participant) and analyzed the data descriptively.

We assessed students’ overall attitude toward the course with a 16-item evaluative survey consisting of 11 multiple-choice, one slider scale, and four free-response items. The survey was created by SV, who has a Master’s in Education, had advanced training in survey design and experience in qualitative research, and was the course director, all providing content validity evidence. We read survey items aloud among the author group and piloted the survey with a small reference population to optimize response process validity. The survey was distributed on the last day of class (Appendix E). We calculated and reported descriptive statistics for survey questions with discrete answer choices. For free-response data within the survey, two authors (SV and AV) performed a thematic analysis. SV trained AV, a senior resident, to perform a thematic qualitative analysis. The analysts independently reviewed the data and later met to establish a final coding scheme, which they then independently applied to all data. After applying the final coding scheme, they identified discrepancies and finalized themes. The simple percent agreement between the two analysts was 80.3%. Discrepancies were resolved via in-depth discussion and negotiated consensus.

This study was deemed exempt by the University of California, Los Angeles Internal Review Board (IRB #20-002014) approved on November 19, 2020.

## IMPACT/ EFFECTIVENESS

Twenty-six students participated in the virtual clerkship representing 22 medical schools and all regions of the US. All students completed the medical knowledge pre- and post-test. Mean test scores improved from 21.5 (standard deviation [SD] +/−2.6) to 22.9 (SD +/− 1.24) (*P* = 0.006), effect size 0.68, 95% confidence interval, 0.12–1.24.

Of the 130 IDHEAL post-module surveys administered, 98 (75%) were completed. Of the modules chosen, incarceration was least likely to have been previously covered with only 6% (1/18) of respondents having prior instruction. Eighty-nine percent (87/98) of respondents “strongly agreed” that these topics were important for patient care in the ED, and 66% (65/98) felt more confident after completing the modules. See Table 2 for full results.

Almost all (25/26, 96%) students completed the end-of-rotation evaluative survey. Of all respondents, 95% “strongly agreed” or “agreed” that the rotation should be repeated in the future, and all “strongly agreed” or “agreed” that the rotation would impact the way they ranked our program. Major themes from the qualitative analysis are described in Table 3.

Prior literature has demonstrated knowledge acquisition and retention from virtual curricula, and we saw similar results, albeit our study demonstrated only a modest improvement.^35^,^36^ One explanation for the lack of larger change is that our assessment items may not have been perfectly aligned with our curriculum as the questions were pulled from a standard question bank. For future versions, we would strongly consider constructing and validating our own internal assessment of medical knowledge to be better aligned with our objectives. Additionally, students rated themselves as more confident in discussing and managing social medicine topics. Ideally, we would be able to conduct a repeat assessment at a predetermined timepoint to assess whether the social EM content had modified their practice as residents. While we emphasized our program’s strong social EM vision, other programs may replicate this curriculum to focus on their own strengths. In the past, visiting clerkships have acted as a recruiting tool for residencies,^8^ and virtual clerkships may also allow residencies to highlight strengths and successfully recruit.

Students from 22 institutions participated in our clerkship at minimal cost to them (only the cost to apply via VSAS). In contrast, EM applicants averaged 1.9 visiting rotations costing almost $1000 per rotation in 2019.^37^–^39^ While many institutions have implemented scholarships for under-represented in medicine students, virtual clerkships remove financial barriers for all students and may be an invaluable option for students with familial or other obligations.^40^ Virtual clerkships represent an additional strategy to help mitigate the socioeconomic barriers of visiting rotations.

While students perceived our virtual experience to be valuable, several challenges were encountered. The clerkship required significant administrative efforts and a large number of facilitators to create the intimate small-group experiences critical to its success. There was no protected time or funding for the instructors. Overall, at least 40 hours per clerkship among NW and SV were required, which did not account for planning and time from all instructors who volunteered their time. These requirements may be adjusted by limiting the number of students enrolled. Furthermore, as we look to the future, simultaneously administrating a virtual clerkship and in-person clerkship will likely require significant additional administrative support.

While this rotation ultimately served 25 students, we considered this rotation to be a success as we were fortunate to match three interns of our current class from the Virtual Clerkship. If we were to repeat this clerkship again, we would expand our evaluation efforts as it was limited, mainly only allowing for assessment in small groups. One other addition would be to encourage asynchronous communication among students and faculty. Examples of communication would be continued improvement of the environment and incorporation of daily questions expanding on the day’s content to further enhance spaced repetition. Lastly, interest in our virtual clerkship was likely increased due to COVID-19 restrictions on in-person opportunities. Future versions will require more advertisement and may not bolster as much interest.

We envision we will offer both versions of each clerkship separately moving forward. However, we likely would not offer a formal SLOE to students who pursue the Virtual Clerkship given we cannot comment on their clinical skills in the virtual format. However, we would gladly write a letter of recommendation as, in some ways, program leadership may get to know these students in a more personal way, especially with certain aspects of the SLOE such as “commitment to EM.” Finally, we may incorporate some of the social EM content and other teaching modalities into the traditional clerkship.

## CONCLUSION

This virtual clerkship was created in response to an acute educational need created by the COVID-19 pandemic. However, our experience suggests that virtual learning experiences may be valuable in the future as an adjunct to traditional in-person rotations. Virtual rotations provide flexibility allowing for the incorporation of topics not traditionally taught (eg, social EM), allow residencies and students increased access to one another, and may eliminate socioeconomic barriers advancing educational equity.

## Supplementary Information



## Figures and Tables

**Table 1:** Course goals and objectives of a virtual emergency medicine clerkship.

Goals	Objectives
To build upon existing EM knowledge through less commonly taught core EM chief complaints To expose students to the broad variety of ED practice environments and patient populations they will care for through panels and case-based discussions To improve the knowledge base, and importance of justice in healthcare in caring for ED patients of diverse socioeconomic statuses, race, ethnicities, gender, and sexual orientations. To introduce students to clinical and non-clinical niches in EM including toxicology, critical care, ultrasound, EMS, medical education, research, healthcare administration, and social determinants of health To expose students to a variety of learning modalities including practicing their own teaching skills To introduce the concept of professional skill-set development and how growth mindset may impact clinical encounters	By the conclusion of this rotation, the students should be able to: Medical Knowledge 1. Describe an approach to several commonly seen chief complaints in EM. Social EM 2. Compare how different practice environments, associated healthcare systems, and access to care affect care plans. 3. Discuss areas within medicine, including within EM, how biases may affect patient care and create strategies to overcome one’s own bias. 4. Describe how language, race, gender, homelessness, and addiction affect patient care. Professional Identity Formation 5. Describe clinical and non-clinical practice environments as well as niches within EM. 6. Understand the importance of a growth mindset over a fixed mindset and develop strategies to incorporate a growth mindset. 7. Apply principles of growth mindset to commonly experienced scenarios in the clinical setting. 8. Describe challenges of interviewing virtually. 9. Outline effective strategies for identifying medical content, learning, and organizing medical knowledge in the 21st century. 10. Describe challenges and opportunities of teaching in the 21st century. 11. Demonstrate ability to teach peers on pre-selected topic.

*EM*, emergency medicine; *ED*, emergency department; *EMS*, emergency medical services.

**Table 2:** International and Domestic Health Equity and Leadership sessions survey.

Question/statement	Yes	No			
Have you ever had formal instruction on social determinants of health? (n = 98)	81 (83%)	17 (17%)			
Have you ever had formal instruction on social determinants of health during an emergency medicine rotation or departmental education conference? (n = 98)	42 (43%)	56 (57%)			
Have you ever had formal instruction on topic discussed					
Topic (n = 98)	Yes	No			
Language (n = 23)	8 (35%)	15 (65%)			
Incarceration (n = 18)	1 (6%)	17 (94%)			
Gender (n = 16)	9 (56%)	7 (44%)			
Homelessness (n = 15)	6 (40%)	9 (60%)			
Race (n = 16)	11 (69%)	5 (31%)			
Unknown (n = 10)	7 (70%)	3 (30%)			
Please rate your agreement with the following statement	Strongly agree	Agree	Neutral	Disagree	Strongly disagree
I learned about how this topic affects the health of my patients.	79 (80%)	18 (18%)	0 (0%)	0 (0%)	1 (1%)
I feel more confident about how to address this topic when seeing patients in the ED.	65 (66%)	28 (29%)	4 (4%)	0 (0%)	1 (1%)
This topic is important for the care of patients in the ED.	87 (89%)	10 (10%)	0 (0%)	0 (0%)	1 (1%)

*ED*, emergency department.

**Table 3:** Themes identified from the end-of-rotation survey.

Domain	Themes	Exemplar quotes
Favorites		
Course design	Interactive education	Respondent 11: “Some of my favorite sessions were the teaching sessions, the escape room, and the simulation” Respondent 12: “board style cases from foundations” Respondent 23: “Enhancing my medical knowledge/clinical skills by participating in numerous simulations and mock oral board-style, small group exercises”
	Topic variety	Respondent 9: “Incredible mix of content and social EM” Respondent 10: “Having this diversity of sessions made it easier to engage fully for the two hours that each topic was covered every day. It would’ve been tough to stay focused for 4 hours of medical knowledge didactics or 4 hours of social EM/career topics, so mixing it up was great” Respondent 20: “I like the mix of medical content with professional development and social issues.”
	Social determinants of health focus	Respondent 6: “The social EM aspect of this course was incredibly powerful and important” Respondent 11: “Social discussions are incredibly valuable and vital to the work providers (particularly in EM) do. The medical knowledge will always be hammered into us whether it’s in medical school or residency, but the social determinants of health are truly vital in understanding the populations we serve.” Respondent 12: “Learning about social aspects of EM from experts and listening to their experiences and perspectives”
Professional identity formation	Networking with other students, residents, faculty	Respondent 10: “it was a great way to get to know more about my peers and build a bond.” Respondent 11: “I loved meeting other students from across the country.” Respondent 12: “I absolutely loved how many attendings, residents, and fellows I was able to meet. If I came there in person. I do not think I would have met even an eighth as many.”
	Exposure to future career opportunities	Respondent 4: “it was great to get to know people who specialize in different areas and options for fellowship” Respondent 18: “information about the program, fellowships and culture”
Least favorite/barriers		
Technology related	Screen time	Respondent 1: “long zoom hours” Respondent 7: “Towards the end of the last week I was feeling the zoom fatigue”
	Technical issues	Respondent 3: “Zoom challenges can be rough audio and freezing” Respondent 5: “only issue were the brief problems with Wi-Fi connectivity”
Course design	Too much content for time allotted	Respondent 14: “I wish we had a bit more time on the foundations cases or had a follow up 10-15 minute review of the topics” Respondent 15: “I wish there was a little more small group time!”
	Instructor orientation	Respondent 4: “Whenever you split people into small groups, ALL proctors should implement the round-robin approach for participation AND the proctor should tell the students when their turn is over. Most proctors did this, and I really appreciate it. When it didn’t happen, the sessions felt less fluid”
	Alignment of asynchronous and synchronous content	Respondent 9: “Some of the asynchronous resources were not too connected to the sessions that day.”

*EM*, emergency medicine.
