# Supplementary material for: An Emergency Medicine Virtual Clerkship: Made for COVID, Here to Stay
Source: West J Emerg Med. 2021 Dec 17;23(1):33–9. doi: 10.5811/westjem.2021.11.54118 (PMC8782130; doi:10.5811/westjem.2021.11.54118)
Supplement: Supplementary file 1 [file wjem-23-33-s001.docx]

WestJEM: An Emergency Medicine Virtual Clerkship: Made for COVID, Here to Stay

**Appendix A: Virtual Escape Room Activity “Escape the Toxin”**

*Welcome to our Virtual Escape Room,
Where we're looking for the toxin that's been consumed.
We're so glad you came because we've been trapped
And we have a mystery that needs to be unpacked!
We've only got 45 minutes for this case
And lots of clues we need to trace.
It's open book, open internet so no need to fret,
You can also ask your friends if you forget.
Just fill out this form to see what's next,
Okay, ready, set, go, let's see whose best!*

1. Please enter the date:

2. Please enter your start time:

3. Team Name:

4. What are the names of all the team members?

5. What's the name of your facilitator?

**Part 1.**

*Oh no! EMS rushes in with a mystery that they need you to solve. A 24 yo male has been found down at Santa Monica Pier.*

What is your first step?

*Mark only one oval.*


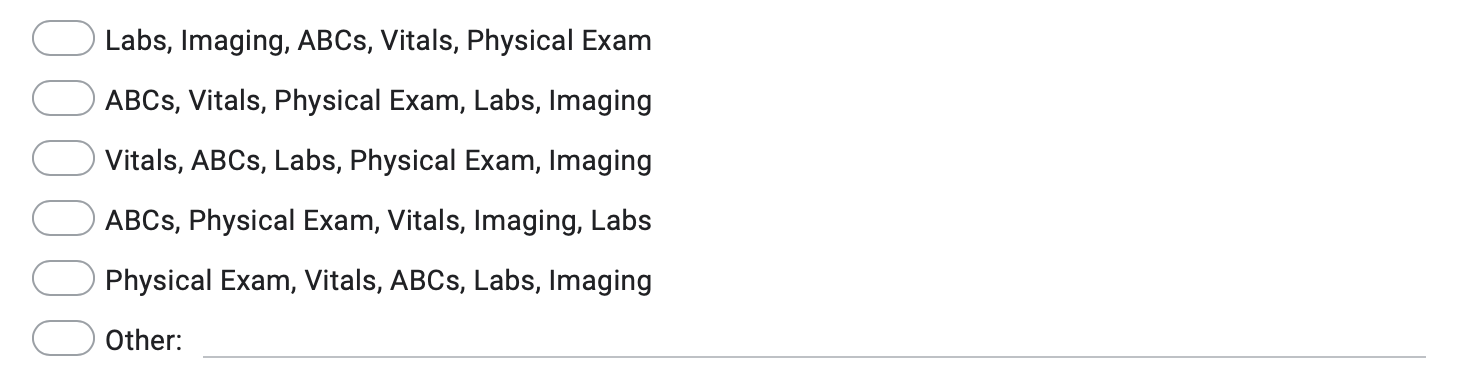


**Part 2.**

*You notice the patient is babbling nonsense and flailing his limbs. When you listen to his lungs you hear breath sounds on both sides and he has strong pulses on both wrists and feet. Whew! What a relief! ABCs are intact.

You ask to have the patient placed on the monitor and his vitals are as follows:
HR 125
BP 96/54 (MAP 68)
RR 22
PO2 98% (on room air)
POCT gluc 128*

It sure is a mystery when you can't take a history... Solve the following word scramble on the differential that could be causing this altered state and you'll receive a key to help you through this misery. (Once you finish the word scramble, your facilitator will provide a CAP-sensitive password to enter so you will receive points for this question.)


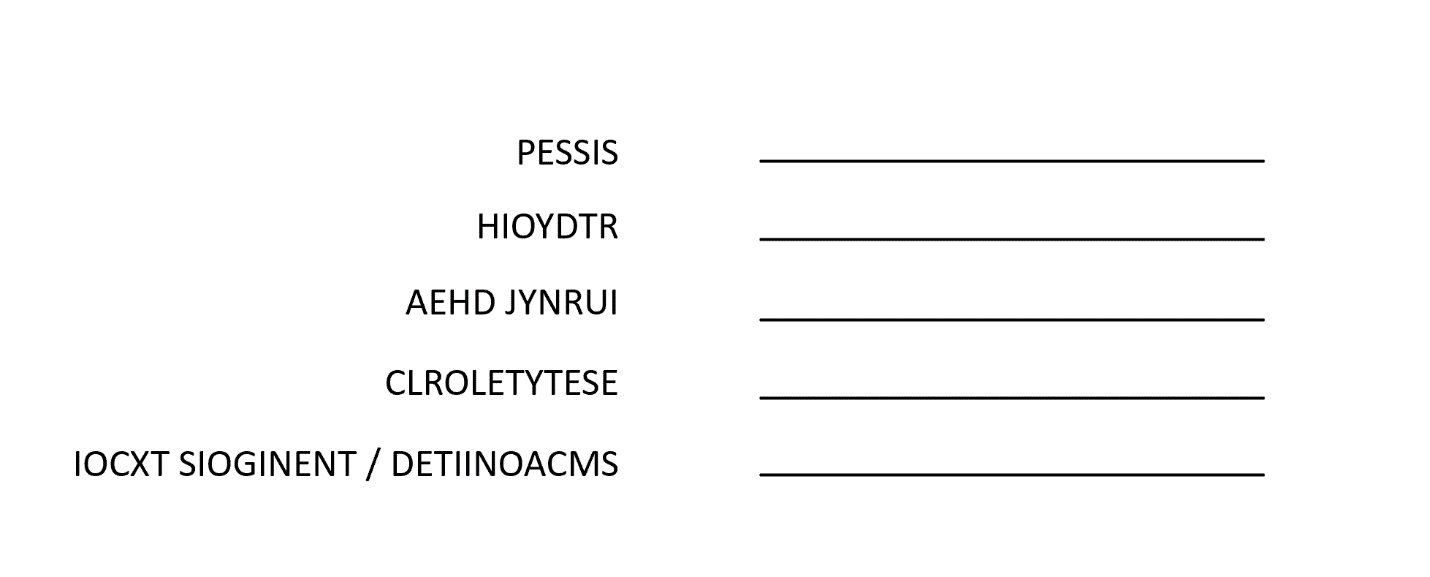


**Part 3.**

*Let's get physical!

HEENT: Atraumatic, normocephalic, no scalp hematomas or lacerations, pupils dilated bilaterally, reactive to light, dry mucous membranes
Neck: In C-spine collar, no step-offs
CV: Tachycardiac, palpable peripheral pulses, regular rhythm, no M/G/R
Chest: No crepitus, no clavicular deformity
Pulm: CTA bilaterally, equal BS bilaterally, no crackles/wheezes/rhonchi
Abd: Soft, NTND, no surgical scars
GU: no perineum lacerations
MSK: Muscle rigidity, no obvious deformities
Skin: Very warm to touch, dry
Neuro: AAOx0, hyperreflexive, moving all extremities spontaneously and symmetrically. GCS 11 (spontaneously opens eyes, inappropriate words, withdrawals from pain)*

You exam the patient and everything does not seem quite right... what's concerning you on that physical exam?


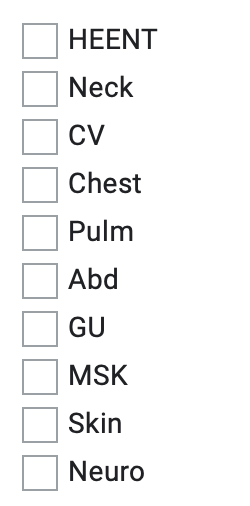


**Part 4.**

*Is it getting hot in here? Maybe we should take off all his clothes...*

We may be missing a vital sign... solve this math puzzle to figure out exactly how hot it's getting.  * (*Mark only one oval.)*


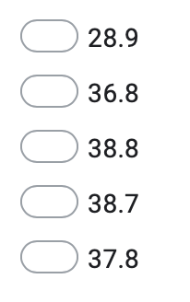


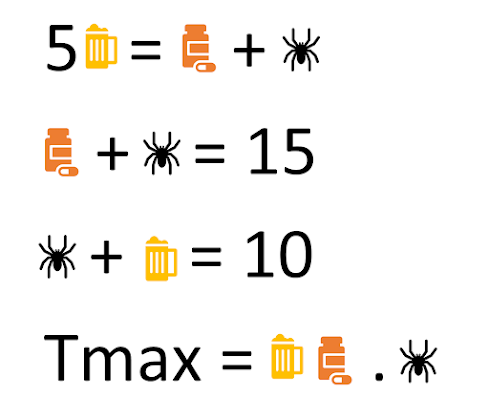


**Part 5.**

*Oh my, what a predicament! That temperature is quite elevated! Plus those dilated pupils, hyperreflexia, muscle rigidity, and dry skin...*

Pick all options that could cause this clinical picture:

Picture A: Jimson Weed

Picture B: Picture of Benadryl

Picture C: Pesticides

Picture D: Picture of Haldol

Picture E: Picture of Amitriptyline

Picture F: Picture of Crystal Meth

**Part 6.**

*Finally you've caught a break! The patient's partner arrives in the hospital to give you some background information. He says his partner only has one medical problem for which he takes medication. Unfortunately for you, the partner is a Lit major and decides the best way to convey this information is through verse:*

The day is cold, and dark, and dreary;
It rains, and the wind is never weary;
The vine still clings to the mouldering wall,
But at every gust the dead leaves fall,
And the day is dark and dreary.

My life is cold, and dark, and dreary;
It rains, and the wind is never weary;
My thoughts still cling to the mouldering Past,
But the hopes of youth fall thick in the blast,
And the days are dark and dreary.

Be still, sad heart! and cease repining;
Behind the clouds is the sun still shining;
Thy fate is the common fate of all,
Into each life some rain must fall,
Some days must be dark and dreary.

- Henry Wadsworth Longfellow

Hmmm... now that the picture is a little bit clearer, we need to start to consider what we need to help this get us through this thriller. Discuss in your small group what labs, imaging, and interventions you would order for this patient. (Once done, your facilitator will provide a CAP-sensitive password to enter so you will receive points for this question.)

**Part 7.**

*Oh no, your nurse hands you the ECG and the attending is no where to be found. You got this!*

Typical ECG showing TCA toxicity

What is the approximate rate?

*Mark only one oval.*


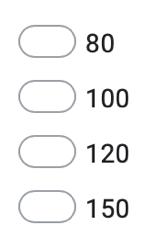


What is the rhythm?

*Mark only one oval.*


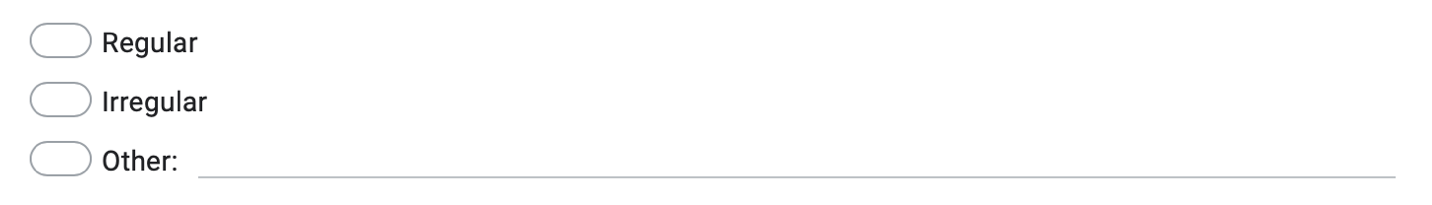


What's the axis?

*Mark only one oval.*


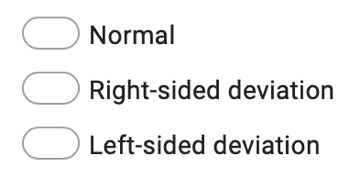


What are the intervals?

*Mark only one oval.*

**
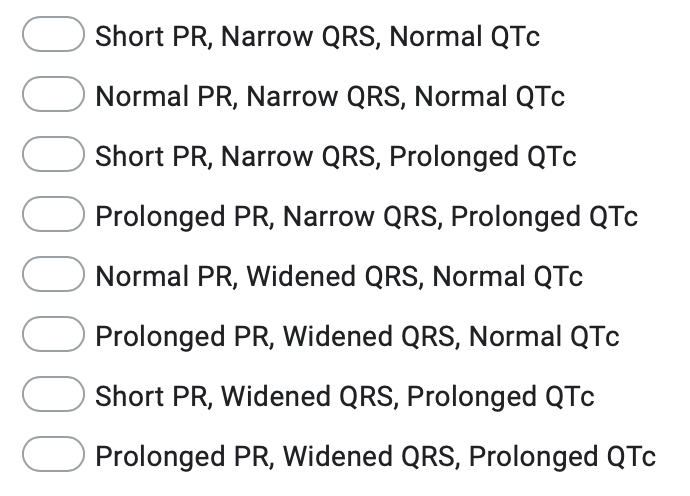
**

**Part 8.**

**Gulp.* You've got a prolonged QRS and a prolonged QTc in your hands. "Hey doc, what do you wanna do now?"*

Same ECG as Part 7

What is the best treatment for the QRS prolongation in this scenario?

*Mark only one oval.*


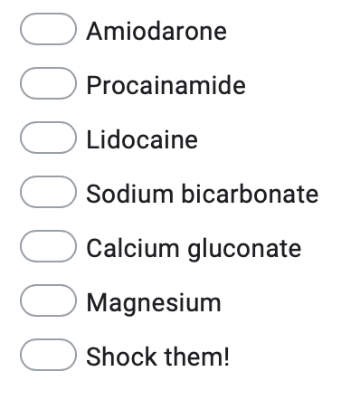


What is the best treatment for QTc prolongation?

*Mark only one oval.*


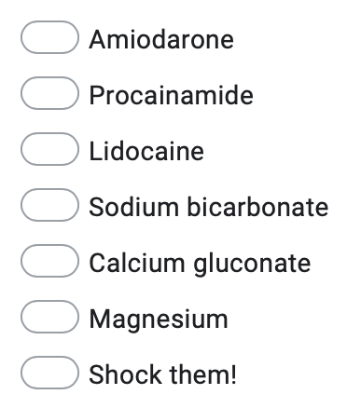


**Part 9.**

*What a pro! After giving bicarb, it's clear those brains are not all for show. Here's an updated ECG after bicarb administration. It also looks like your labs are returning and Mr. Mystery is heading to the CT scanner!*

Improved ECG


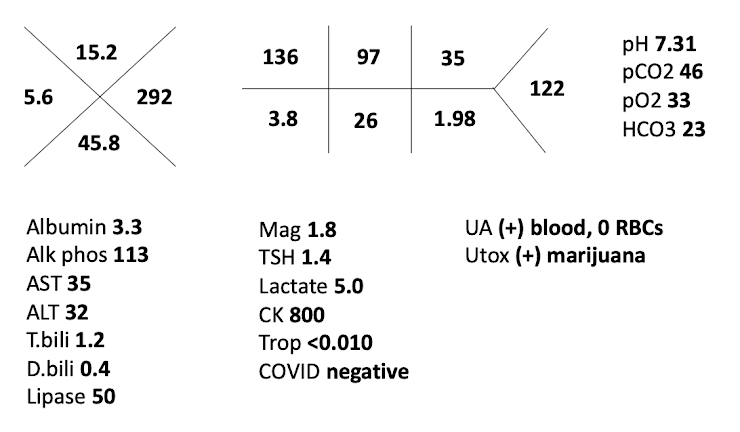


Single slice of a Normal Head CT

Hey doc, what do you think of the head CT? *

*Mark only one oval.*


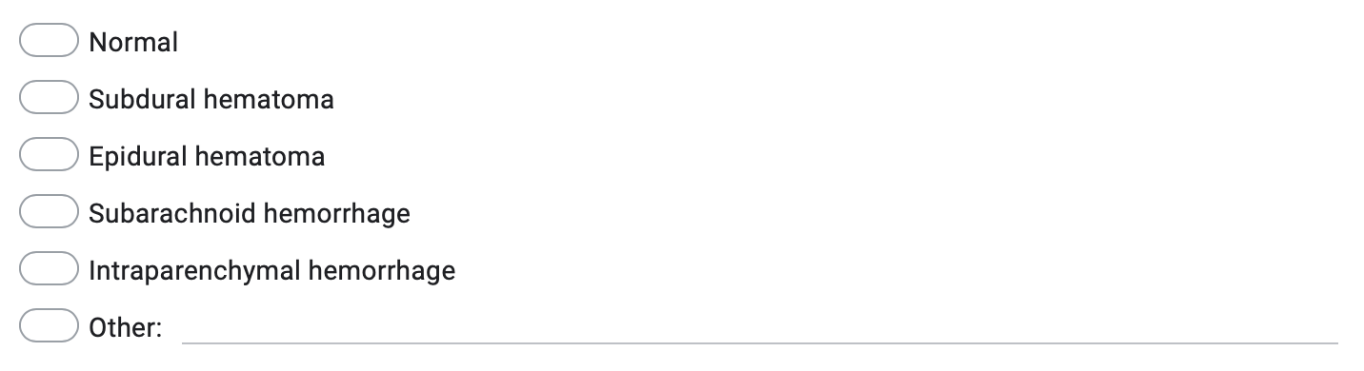


**Part 10.**

*"Doc! Something is happening in CT scanner 1!"*

Solve the following word puzzle to find out what the heck is going on as you run to the scanner...


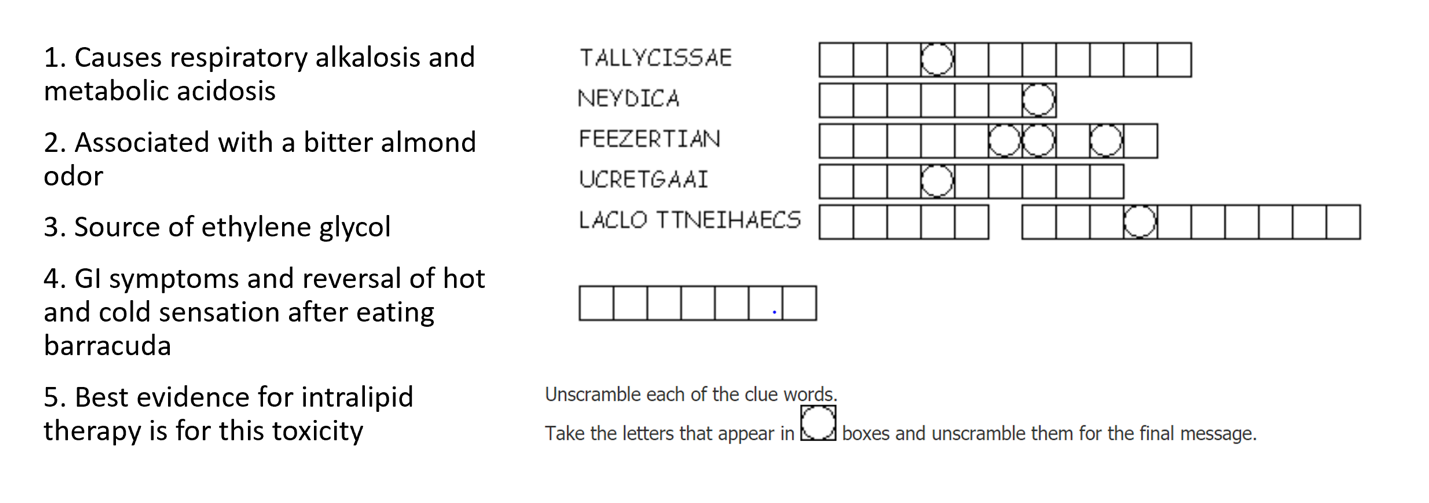


**Part 11.**

*Oh goodness, Mr. Mystery is causing a ruckus in the scanner. He's almost shaking more than you are as you decide what meds to give...*

What's the first line treatment for seizures?

*Mark only one oval.*


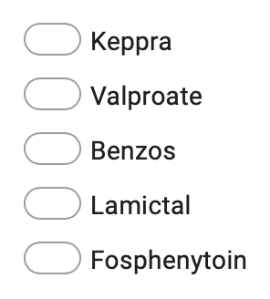


Uh oh. It's been 5 minutes and Mr. Mystery is still seizing despite your first line treatment. Which of these would be a good second line treatment? Check all that apply


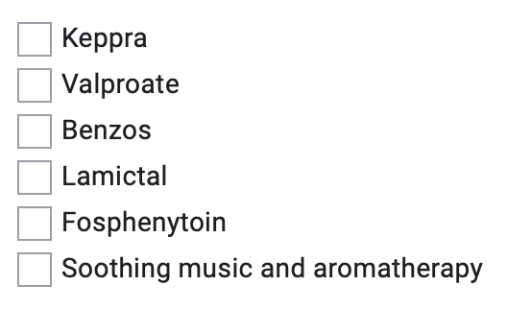


**Part 12**

*Given the seizures and the concern that Mr. Mystery will not be able to protect his airway, you have decided to intubate. Time to grab the airway cart!*

Picture of a “Crash”/Airway cart

Oh no, you run to the airway cart to get supplies but it's locked and you don't remember the passcode. Ugh! If only you'd paid attention during orientation when you were getting a tour of the ER. Solve this puzzle to figure out the code to unlock the cart:


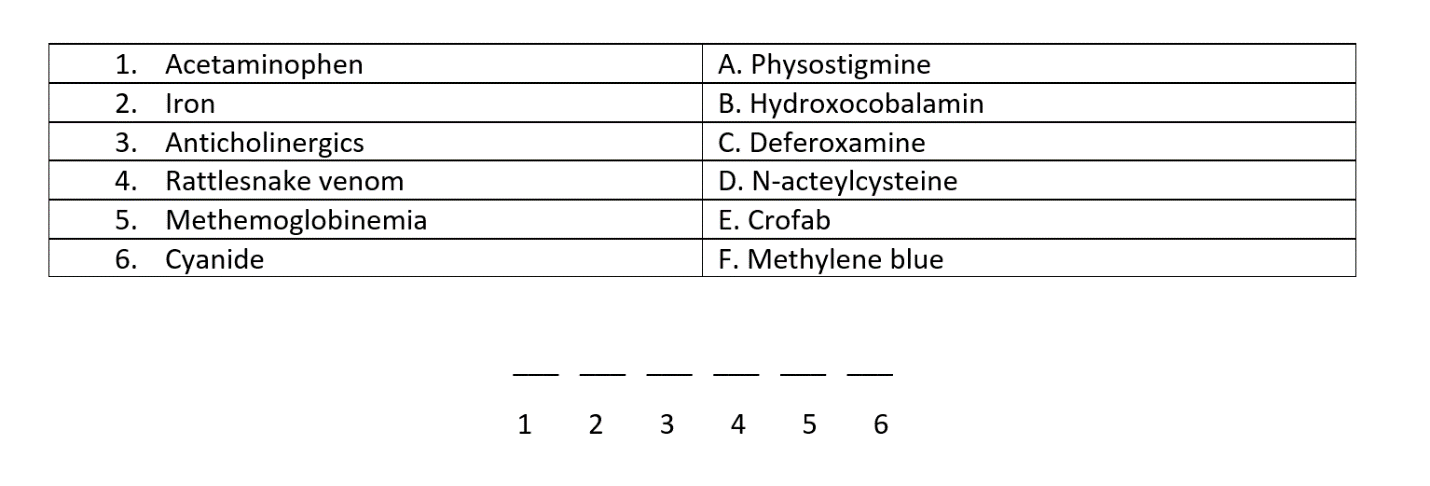


**Part 13.**

*Great job! You've successful completed a first-pass intubation of Mr. Mystery and suppressed his seizures. Now what?*

What is the best disposition for Mr. Mystery?

Picture A: Picture of Santa Monica

Picture B: Picture of a hospital room

Picture C: Picture of a hospital hallway

Picture D: Picture of an ICU room

**Part 14.**

*Way to go! We're halfway there!
Just kidding... 2 more questions to go.*

It might be clear, but please be a dear, if it's not too much trouble, here's one last puzzle.


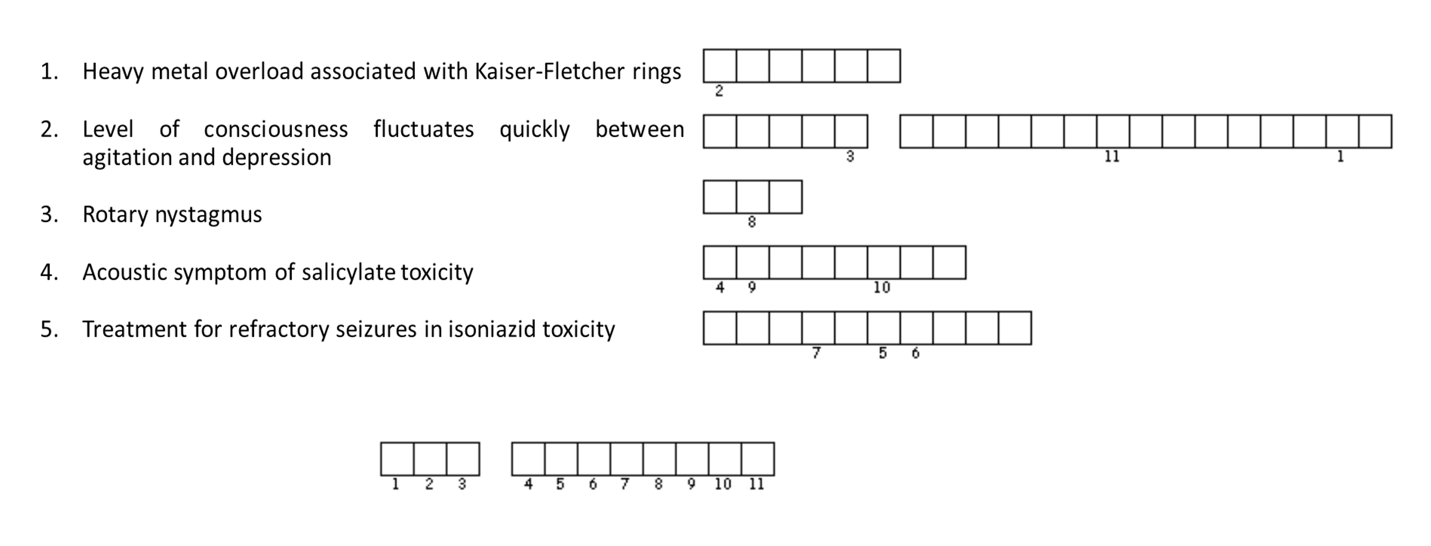


**Part 15.**

*Woohoo! You figured it out! Tricyclic Antidepressants!*

Discuss - what is the mechanism of sodium bicarb for TCA toxicity?

**The End!**

*Thank you so much for playing along! We would love if you gave us some feedback through the link sent to you on what you thought about this activity!*

Please enter the end time:

WestJEM: An Emergency Medicine Virtual Clerkship: Made for COVID, Here to Stay

Appendix B: Small Group Instructor Expectations

**
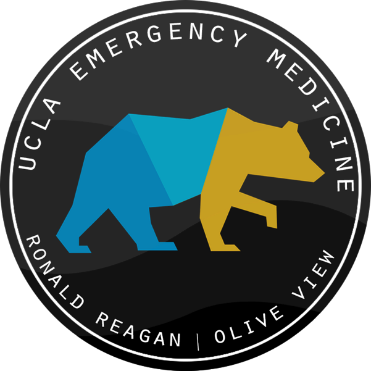
Tips for Teaching Small Groups on Zoom**

Welcome to the brave new world of facilitating small groups on Zoom! We’ve compiled these tips to help you navigate these new waters to hopefully better engage your students on this new platform.

- Set expectations for participation at the beginning of your session 🡪 we will also do this during orientation but it will help to re-enforce it during each session
  - Video must be on
  - Discuss the goal of approximately equal time participation for each student 🡪 this will hopefully encourage less vocal students to participate more and more vocal students to share the floor with their other classmates
  - Let them know their evaluation is almost entirely based on participation, communications and being respectful of each other in small groups rather than having “the right answer” 🡪 these small groups are meant to be a safe learning space
  - Students can always “phone a friend” if they don’t know the answer to something
- Start the session with a quick introduction of everyone and consider including a personal fact, question etc. so students can get to know each other and you better
- Consider the phrasing, “Student X answered the last question, does anyone else have any suggestions for next steps in the case?” This signals to student X that they should yield the floor and encourages the other students to engage.

WestJEM: An Emergency Medicine Virtual Clerkship: Made for COVID, Here to Stay

**Appendix C: Example Schedule**

Week 1Example


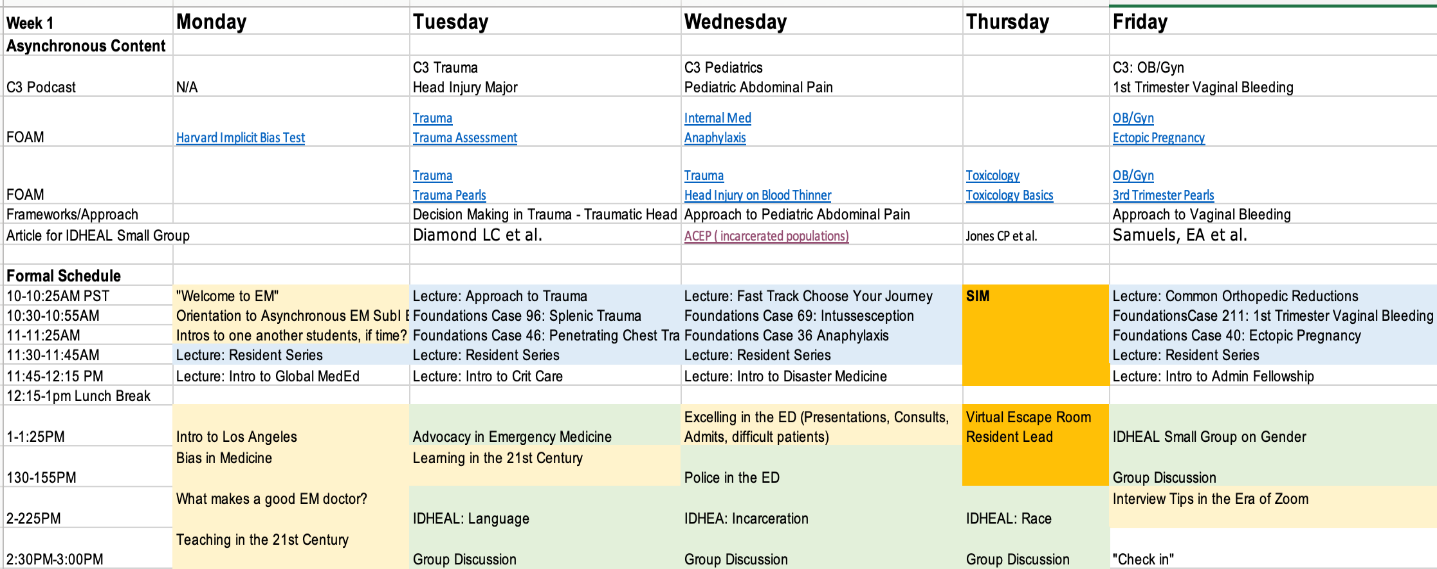


Week 2 Example


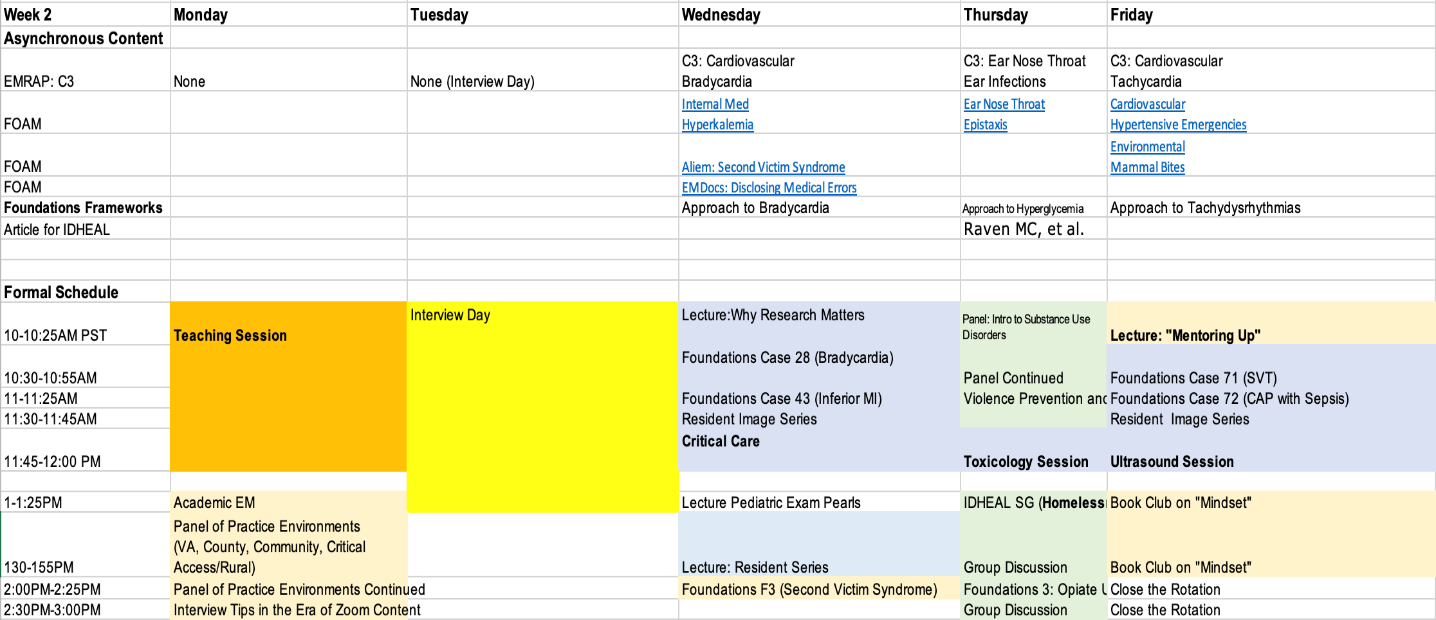


**Appendix C.** Course schedule. Example Schedule Template: Blue (Social EM), Yellow (Professional Development/Professional Identity Formation, Purple (Career Opportunities), White (Medical Knowledge), Orange (Virtual Simulation and Teaching Session), Green (Virtual Escape Room.

EMRAP C3: Listed by subject

Foundations Cases: Listed by specific case

Foundations Frameworks: Listed by specific frameworks

Lecture: Resident Series: 15 minute lectures given by residents

WESTJEM: An Emergency Medicine Virtual Clerkship: Made for COVID, Here to Stay

**Appendix D: IDHEAL surveys**

IDHEAL Social EM Teaching Modules Feedback Survey

1. What is your level of training?
   MS3 MS4 PGY1 PGY2 PGY3 PGY4 NP Attending other
2. Have you ever had formal instruction on the topic discussed today?

Yes No

1. Have you ever had formal instruction on social determinants of health?

Yes No

1. Have you ever had formal instruction on social determinants of health during an emergency medicine rotation or departmental educational conference?

Yes No

1. During which part of your shift was this session taught?

Teaching session after sign-out, Bedside teaching, Impromptu teaching session

1. Which topic was covered during this session?

Gender, Housing Insecurity Employment Human Trafficking Health Literacy Medico-Legal Healthcare Coverage Built Environment Language Debt Immigration Financial Stress Transportation Education Culture Homelessness Food Insecurity Race Incarceration Violence

**Please answer to what degree you agree with the following statements:**

7.   I learned about how this topic can affect the health of my patients.

Strongly disagree, Disagree, Neutral, Agree, Strongly Agree

1. I feel more confident about how to address this topic when seeing patients in the emergency department.

   Strongly disagree Disagree Neutral Agree Strongly Agree
2. This topic is important for the care of patients in the ED.
    Strongly disagree Disagree Neutral Agree Strongly Agree
3. Think about the patients you have seen in your past 3 ED shifts. Please estimate the relative contribution of each type of emergency to the ED visits of the patients you have cared for. (Total percent should add to 100)

a. Biomedical_____% b. Social______% c. Psychologic_____%

11. Did you learn something that will change what you do on shift today?

Yes No

If Yes, please state what you will change:

Free Text

Please provide any additional comments or feedback below:

Free Text

WestJem: An Emergency Medicine Virtual Clerkship: Made for COVID, Here to Stay

**Appendix E: Post Rotation Survey**

1. Please rate your agreement with the following statement: The time required to complete the asynchronous content was appropriate.

Strongly agree, Agree, Neither agree or disagree, Disagree, Strongly disagree

1. Please rate your agreement with the following statement: The asynchronous content was helpful in the “in-person” sessions.

Strongly agree, Agree, Neither agree or disagree, Disagree, Strongly disagree

1. Please rate the difficulty level of the foundations small group sessions.

Very easy, Easy, Neither easy nor difficult, Difficult, Very difficult

1. Please rate your agreement with the following statement: This virtual clerkship should be repeated in the future.

Strongly agree, Agree, Neither agree or disagree, Disagree, Strongly disagree

1. The Virtual Escape Room was an effective way to teach cases.

Strongly agree, Agree, Neither agree or disagree, Disagree, Strongly disagree

1. The "Teaching session" was a worthwhile experience and should be repeated in the future.

Strongly agree, Agree, Neither agree or disagree, Disagree, Strongly disagree

1. The simulation session was a worthwhile experience and should be repeated in the future.

Strongly agree, Agree, Neither agree or disagree, Disagree, Strongly disagree

1. The Virtual Book Club was a worthwhile experience and should be repeated in the future.

Strongly agree, Agree, Neither agree or disagree, Disagree, Strongly disagree

1. This rotation will have an impact in the way I rank this program

Strongly agree, Agree, Neither agree or disagree, Disagree, Strongly disagree

1. I enjoyed meeting people from across the country

Strongly agree, Agree, Neither agree or disagree, Disagree, Strongly disagree

1. What is one thing Rosh Review can do to improve its Qbank?

Free text

1. How likely is it that you would recommend Rosh Review to your fellow residents

Scale 1-100

1. Even in a non-COVID era, this rotation should be continued into future calendar years

Strongly agree, Agree, Neither agree or disagree, Disagree, Strongly disagree

1. What was my favorite thing about this rotation?
2. What was my least favorite thing about this rotation?
3. General Comments
